# Editorial Note: Recruitment of a SAP18-HDAC1 Complex into HIV-1 Virions and Its Requirement for Viral Replication

**DOI:** 10.1371/journal.ppat.1014251

**Published:** 2026-05-26

**Authors:** The PLOS Pathogens Editors

The *PLOS Pathogens* Editors issue this Editorial Note to update readers that all blot panels in the upper six right-hand panels of Fig 1A and in Fig 3A of [1] and in Fig 2B of [2] are derived from the same sequentially-probed single experimental replicate blot, with the data re-used in [1] to represent some of the same results and reporting the same experimental conditions as previously reported in [2].

Specifically,

- In Fig 1A, the top right-hand INI1, BRG1, and BAF 155 panels are from the same blot as lanes 1–2 and 7–8 in the INI1, BRG1, and BAF 155 panels, respectively, of Fig 3A.
- In Fig 1A, the top right-hand INI1 and gp41 panels are from the same blot as lanes 1–2 and 7–8 in the INI1/hSNF5 and gp41 panels in Fig 2B of an earlier publication by some of the same authors [2].
- The INI1 panel in Fig 3A is from the same blot as the INI1/hSNF5 panel in Fig 2B of an earlier publication by some of the same authors [2]

The text in the first paragraph of the Results section of [1] indicates that the INI1 results in Fig 1A of [1] are consistent with the previous reported findings in [2]. Upon follow-up, the corresponding author stated that images in the upper six right-hand panels of Fig 1A and in Fig 3A were generated from re-probing the same blot as reported in Fig 2B of [2] to conserve samples. They stated that a fresh set of antibodies was used to sequentially re-probe the blot for INI1, p24 and gp41, as well as probing for additional protein targets using anti-BRG1, anti-BAF155, anti-BAF170, anti-SAP18, anti-SAP30, and anti-HDAC1. The left-hand panels and lower INI1 and hBRM panels of Fig 1A represent new blots.

The top right HIV-1_MN_ – INI1 and gp41 panels of Fig 1A and the INI1 panel of Fig 3A in [1] were previously published by an overlapping author group [2]; Copyright © 2004 American Society for Microbiology. The American Society for Microbiology granted permissions for this content to be republished in *PLOS Pathogens* under a CC BY license. Please provide due attribution to the original publication when referring to this content.
